# Myocardial revascularization using on-pump beating heart among patients with left ventricular dysfunction

**DOI:** 10.1186/1749-8090-5-109

**Published:** 2010-11-10

**Authors:** Ahmad K Darwazah, Vivian Bader, Ismail Isleem, Khalil Helwa

**Affiliations:** 1 Department of Cardiac Surgery, Makassed Hospital, Jerusalem, Israel; 2 Department of Cardiology, Makassed Hospital, Jerusalem, Israel

## Abstract

**Objectives:**

On-pump beating heart technique for myocardial revascularization has been used successfully among both low and high risk patients. Its application among low ejection fraction patients is limited. The aim of our study is to evaluate this technique among patients with low ejection fraction and to compare results with off-pump bypass technique.

**Methods:**

This retrospective study includes 137 patients with ejection fraction below 0.35 who underwent isolated coronary artery bypass surgery. 39 patients underwent myocardial revascularization using on-pump beating heart (ONCAB/BH), while 98 patients had off-pump beating heart (OPCAB). Different preoperative, operative and postoperative variables were evaluated among both groups.

**Results:**

Patients profiles and risk factors were similar among both groups, except for the number of patients undergoing redo CABG which was significantly higher among ONCAB/BH (13% vs 3%; p = 0.025). Ejection fraction (EF) varied from 10-34%. The mean EF for patients who underwent ONCAB/BH was 28 ± 6 in comparison to 26 ± 5 for OPCAB patients (P = 0.093). Predicted risk for surgery according to EuroSCORE was similar among both groups (P = 0.443). The number of grafts performed per patient was significantly more among patients who underwent ONCAB/BH (2.2 ± 0.7 Vs 1.7 ± 0.7; P = 0.002). Completeness of revascularization was significantly greater in the ONCAB/BH patients (72% Vs 46%, P = 0.015). The incidence of hospital mortality and combined major morbidity was more among ONCAB/BH in comparison to OPCAB, but the difference was not significant. However, the incidence of blood loss, ventricular arrythmias, inotropic support, ICU, hospital stay and blood transfusion were significantly greater among patients who underwent ONCAB/BH.

**Conclusions:**

On-pump beating heart technique can be used in myocardial revascularization among patients with left ventricular dysfunction. The technique was found to be associated with better myocardial revascularization when compared with OPCAB technique. However, the incidence of morbidity and mortality was more than OPCAB.

## Introduction

Despite the presence of different pump techniques used in surgical myocardial revascularization, the optimal method used is still controversial. No technique was found perfect to be applied to all patients. Nowadays we are confronted with different categories of patients varying from straightforward low risk cases to complicated ones due to the increase in number of elderly patients with complicated coronary anatomy and impaired left ventricular function.

The use of both conventional cardiopulmonary bypass and OPCAB among patients with impaired LVF proved its efficiency and safety [1,2]. Under certain circumstances, the application of both techniques could not be possible and even harmful to the myocardium[3].

In our present work, ONCAB/BH technique was used to revascularize the myocardium among patients with impaired LVF. The results of such technique was compared to those who underwent off-pump beating heart.

## Patients and Methods

This study was performed retrospectively among 137 patients with isolated coronary artery bypass surgery during the period from 1999-2009. The selection of patients was based initially on their ejection fraction. Those with ejection fraction below 0.35 were included only and divided into two groups according to the technique used during myocardial revascularization. Ninety eight patients underwent OPCAB, while thirty nine patients had ONCAB/BH technique. Patients with combined procedure and those patients who initially underwent off-pump and converted to on-pump beating heart were excluded from the study. The decision to use either technique was made intraoperatively based on hemodynamic parameters.

Patients who could tolerate manipulation of the heart without hemodynamic deterioration to visualise target vessels underwent OPCAB revascularization. Those patients who showed hemodynamic deterioration during manipulation underwent ONCAB/BH.

Different preoperative, operative and postoperative variables were evaluated among both groups. Analysis was performed using statistical software version 13 SPSS (Chicago, IL). Data are expressed as percentages and compared using Fisher exact test. Variables are presented as mean ± standard error using student's t test. Statistical significance was assumed when P value was less than 0.05.

## Surgical Technique

Exposure of the heart was performed through median sternotomy. Full heparinization was used in all patients maintaining activated clotting time >400 s. In patients undergoing ONCAB/BH a standard cannulation of the aorta and right atrium was used. A full cardiopulmonary bypass with normothermia was used. Anastomosis of the grafts to coronary arteries was initially performed distally among all patients. Proximal anastomosis to ascending aorta was performed immediately after finishing each distal anastomosis. The sequence by which coronary vessels were grafted varied from one patient to another. Grafting of the LAD by left internal mammary artery was the first to be performed. However, in some patients with huge hearts, grafting of diagonal followed by RCA and circumflex arteries was usually performed first, leaving the LAD at the end to avoid stretching and kinking of LIMA during rotation of the heart.

Anastomosis was facilitated by the use of both U-shaped stabilizer (Guidant, Indianapolis, IN) and suction stabilizers (Medtronic Octopus III). Revascularization of circumflex or obtuse marginal arteries was performed with the help of Starfish apical positioning device (Medtronic, Inc, Minneapolis, MN). Intracoronary shunts (Medtronic Inc.) were used when needed.

## Results

Preoperative patients profile and risk factors are listed in table 1. There was no differences among both groups, except for the number of patients undergoing redo CABG, which was significantly higher among ONCAB/BH (13% Vs 3%, P = 0.025). Predicted risk for surgical intervention according to EuroSCORE was similar among both groups (P = 0.443).

**Table 1:** Preoperative Demographics and Risk Factors

Variable	On-pump BH (n = 39)	Off-pump BH (n = 98)	P value
Age	58 ± 8	57 ± 10	0.100

Female gender	7 (18%)	14 (14%)	0.826

BMI	27.6 ± 4.5	28.2 ± 4.5	0.564

Family History of CAD	19 (49%)	61 (62%)	0.128

Hypertension	17 (44%)	49 (50%)	0.354

Diabetes mellitus	18 (46%)	45 (46%)	0.929

Current smoker	22 (56%)	63 (64%)	0.362

Dyslipidemia	14 (36%)	41 (42%)	0.598

Obesity	12 (31%)	28 (27%)	0.778

Peripheral vascular disease	3 (8%)	8 (8%)	0.814

Carotid artery disease	5 (13%)	12 (12%)	0.387

Urgent operation	8 (21%)	28 (29%)	0.319

COPD	6 (15%)	15 (15%)	0.753

Redo CABG	5 (13%)	3 (3%)	0.025

Chronic Kidney Disease	6 (15%)	11 (11%)	0.126

Recent Angioplasty	6 (15%)	17 (17%)	0.884

Myocardial Infarction	26 (67%)	65 (66%)	0.600

Heart Failure	12 (31%)	52 (53%)	0.241

Unstable Angina	22 (56%)	43 (44%)	0.082

Stroke	5 (13%)	6 (6%)	0.381

Streptokinase	6 (15%)	5 (5%)	0.126

Clopidogrel	6 (15%)	8 (8%)	0.273

EuroSCORE	14.1 ± 11.0	12.2 ± 12.5	0.443

Ejection fraction (EF) among all patients was below 0.35. It varied from 10-34. The mean EF for ONCAB/BH was 28 ± 6 in comparison to 26 ± 5 for OPCAB (P = 0.093). The incidence of main stem involvement was more among ONCAB/BH patients, but the difference did not reach statistical significance (8% Vs 4%, P = 0.765).

The extent of preoperative coronary artery disease was similar among both groups regarding the involvement of LAD, circumflex, second diagonal and obtuse marginal coronary arteries. The extent of right coronary artery disease was significantly higher among OPCAB (70% Vs 51% P = 0.025). On the other hand, involvement of first diagonal coronary artery was significantly higher among ONCAB/BH (33% Vs 17%, P = 0.035). There was no difference regarding the number of coronary vessels affected whether single, double or triple vessel among both groups (P = 0.396).

There was a significant difference regarding the number of grafts used per patient among both groups (Table 2). ONCAB/BH patients received 2.2 ± 0.7 grafts, while OPCAB had 1.7 ± 0.7 (P = 0.002). The difference was due to more grafting of the right and circumflex coronary arteries.

**Table 2:** Operative Data

Variable	On-pump BH	Off-pump BH	P value
Use of LIMA	30(77%)	73(75%)	0.827

Use of RIMA	1(3%)	2(2%)	0.835

LAD Graft	37(95%)	95(97%)	O.544

RCA Graft	9(23%)	19(19%)	0.581

PDA Graft	4(10%)	4(5%)	0.019

D1 Graft	13(33%)	16(16%)	0.381

D2 graft	9(23%)	26(27%)	0.022

OM1 Graft	3(8%)	3(3%)	0.221

OM2 Graft	1(3%)	0(0%)	0.109

Cx Graft	7(18%)	4(4%)	0.182

Operation Time	4.0 ± 1.0	3.7 ± 1.0	0.033

Number of grafts	2.2 ± 0.7	1.7 ± 0.7	0.002

Complete revascularization	28(72%)	45(46%)	0.015

**Figure 1:**
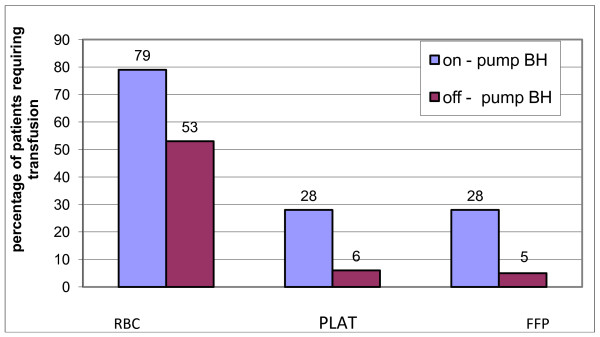
**Percentage of patients requiring blood transfusion and its products**. RBC: Red blood cells, PLAT: Platelets, FFP: Fresh frozen plasma.

Hospital mortality was slightly more among ONCAB/BH patients, but the difference was not significant 8% Vs 6%, P = 0.712(Table 3). The incidence of total major morbidity was more among ONCAB/BH patients, but the difference did not reach statistical significance (P = 0.778). However, the incidence of blood loss, ventricular arrythmias and inotropic support were significantly greater among ONCAB/BH group. Transfusion of red blood cells and its products were significantly greater among ONCAB/BH, P = 0.001 (Figure 1). Postoperative intensive care unit length of stay was significantly higher in the ONCAB/BH patients(35 ± 20 hours vs27 ± 14 hours for OPCAB, P = 0.019). Similarly, postoperative hospital stay was significantly higher in the ONCAB/BH patients (7.1 ± 2.9 days Vs 5.9 ± 2.3 days for OPCAB, P = 0.015).

**Table 3:** Postoperative Morbidity and Mortality

Variable	On-pump BH	Off-pump BH	P value
30-Day Mortality	3 (8%)	6 (6%)	0.712

Morbidity	12 (31%)	26 (27%)	0.778

Infection	4 (10%)	4 (4%)	0.765

Atrial Fibrillation	3 (8%)	8 (8%)	0.959

Ventricular Arrhythmias	9 (23%)	4 (4%)	0.002

Myocardial infarction	5 (13.15%)	8 (8%)	0.192

CVA	1 (3%)	1 (1%)	0.535

Respiratory Failure	3 (8%)	1 (1%)	0.133

Renal Failure	3 (8%)	3 (3%)	0.221

Intra Aortic Balloon pump	7 (18%)	10 (10%)	0.196

Inotropic Support	31 (79%)	29 (30%)	0.001

Estimated blood loss	974 ± 824	548 ± 337	0.001

## Discussion

Conventional non-beating heart on-pump is still the standard technique used in coronary artery surgery. Complications in relation to this technique are due to the release of inflammatory mediators, the use of cardioplegia, aortic cross clamping and hypothermia [4]. Off-pump technique was introduced to avoid such complications. Despite its efficiency and safety over conventional CPB, the technique was criticized by many investigators regarding completeness of myocardial revascularization, graft patency and long term results. One of the important draw backs of this technique is the hemodynamic deterioration which can occur during manipulation of the heart during surgery, which entails urgent transfer to conventional CPB. The results of such surgery proved to be inferior [5].

From our previous study [1] using off-pump bypass among low ejection fraction patients, we found that such a technique is effective in reducing both mortality and morbidity. Nevertheless, we agree with other investigators that the technique is not always associated with complete revascularization. The main obstacle which determines completeness of revascularization is the hemodynamic deterioration which can occur during such a procedure. To avoid such deterioration among our patients, we advocated minimal manipulation during surgery which obviously lead to less number of grafts used and incomplete revascularization.

An intermediatory approach between conventional and off-pump bypass was studied by Perrault and colleagues [3]. They used on-pump beating heart (ONCAB/BH) among their patients with low ejection fraction. They found that using CPB without cross clamping and cardioplegic arrest with the heart beating is associated with less myocardial oedema and ischemia. From their study, they proved that such a technique is effective in preventing myocardial injury and can be effectively used among high risk patients who cannot tolerate cardioplegic arrest or when the use of off-pump is not technically feasible.

Since the work of Perrault, various studies using ONCAB/BH technique for myocardial revascularization among both low and high risk patients was performed [4,6-15]. The technique proved to be a reliable and effective method and was associated with complete revascularization.

The main idea of using ONCAB/BH technique among high risk patients is to avoid the serious manipulation which could be harmful to the myocardium and subsequently to perform complete revascularization. Surprisingly, in our study, we found that the incidence of myocardial infarction was more among patients who underwent ONCAB/BH when compared to OPCAB, which indicates that manipulation of the heart even when supported by the bypass machine is still harmful. In an interesting study performed by Rastan and co-workers[4] using ONCAB/BH among patients with normal ejection fraction, they found an increase incidence of myocardial injury when compared to off-pump. Although, the effect was without any clinical significance they believed that such a technique is not favourable to off-pump bypass. Pegg and co-workers[12], confirmed these findings, by reporting that that the incidence of new *irreversible myocardial injury among patients with impaired LVF was significantly *higher among ONCAB/BH patients when compared with conventional bypass.

The other benefit of using ONCAB/BH is to achieve complete revascularization. Previous studies showed that this technique was associated with adequate number of grafts performed among both low and high risk patients [3,6,9,10,14,15]. Comparing the number of grafts performed to other bypass techniques, conflicting results were obtained. Some studies were in favour of off-pump and conventional bypass over ONCAB/BH [4,7,14], while others were in favour of ONCAB/BH when compared to other techniques of bypass [9]. Prifti and colleagues in their study [11], found a similar number of grafts performed among both conventional and ONCAB/BH. We agree with previous studies that ONCAB/BH technique is associated with adequate number of grafts performed. In our present study, there was a significant difference in the number of grafts performed and complete revascularization was in favour of ONCAB/BH. The main reason for such a difference was due to difficulty in grafting of circumflex and posterior descending coronary arteries among patients undergoing off-pump bypass.

The mortality rate of patients with impaired LVF undergoing ONCAB/BH varies from 2-8% [6,10,11,15]. The difference in mortality among various studies was directly related to the difference in selection of patients. Beside impaired LVF, other associated risk factors were involved, as acute myocardial infarction, cardiogenic shock and patients on dialysis [7,8,15]. In our study, the mortality rate was 7.7%, which was high compared to other studies. The high mortality among our group of patients was related to the impaired left ventricular function. Other factors contributing to the mortality of patients were the preoperative association of heart failure and myocardial infarction, the failure to revascularize both circumflex artery in 15% and RCA in 8% of patients and lower percentage of patients who received LIMA for grafting. Comparing our results with Folliguet and colleagues study[6], they had the lowest mortality among their group of patients despite a similar mean ejection fraction to our patients, we found that the mean EuroSCORE of their patients was 5.8 ± 2.7 in comparison to 14.1 ± 11.0 among our patients. This clearly shows the importance of associated other risk factors affecting mortality beside impaired LVF.

There are limited studies evaluating the incidence of mortality in relation to using either ONCAB/BH or off-pump. Among low risk patients, the mortality rate among patients who had ONCAB/BH was higher than off-pump bypass [9]. On the contrary, in Rastan and colleagues study [4] the mortality rate was more among off-pump bypass patients. In high risk groups, Edgerton and colleagues [8] found that mortality rate was significantly higher among ONCAB/BH patients when compared to off-pump. Similar findings were seen in our study. However, the differences in mortality among our two groups of patients was not significant.

From the above limited studies, it seems that ONCAB/BH is associated with more mortality than OPCAB despite the fact that these patients have adequate myocardial revascularization. The explanation for such unexpected results is related to the use of bypass machine. Early work by Perrault and co-workers [3] found that the release of inflammatory mediators interleukin-6, interleukin-10 and elastase among patients undergoing on-pump BH was not significantly different from conventional bypass. A further study [13] found that ONCAB/BH can trigger an intense inflammatory response, they found that the levels of interleukin-6, interleukin-8, interleukin-10 and tumour necrosis factor-alpha were significantly elevated when compared to off-pump bypass.

It seems that ONCAB/BH technique does not ameliorate the complications encountered with the use of bypass machine and its effect among high risk patients. This is the basic difference from off-pump bypass technique. In our study, the number of patients who had postoperative major morbidity were higher among ONCAB/BH patients when compared to OPCAB technique. The percentage of patients who had postoperative renal failure, infarction, use of inotropic support and IABP were less among off-pump patients. The amount of blood loss and ventricular arrythmias were significantly higher among ONCAB/BH patients. This was reflected on the significant amount of blood transfusion, blood products and longer ventilation time, intensive care and hospital stay.

The application of ONCAB/BH among patients with impaired LVF resulted in conflicting data. Although, patients had significantly better myocardial revascularization, the incidence of mortality and morbidity was more than OPCAB. It seems that ONCAB/BH technique gives a false sense of security believing that the use of bypass machine can protect the heart during manipulation to perform a better revascularization. This could be true among low risk patients, but the scenario is different when the technique is applied to high risk group.

The present study is one of few studies comparing two technique used in myocardial revascularization among patients with impaired LVF. The study carries several limitations, being a retrospective study among a small number of patients, which made the validity of the clinical results limited. Further studies are needed in particular to follow up these patients to find out the benefit which was achieved by increasing myocardial revascularization among patients who underwent on-pump beating heart.

In conclusion, we believe that ONCAB/BH can be used in myocardial revascularization among patients with left ventricular dysfunction. The technique was found to be associated with better myocardial revascularization, more morbidity and mortality when compared to off-pump bypass.

## Competing interests

The authors declare that they have no competing interests.

## Authors' contributions

AKD Performed operations, conception and study design. VB Assist in surgical procedures and acquisition of data. II and KH investigations and follow up of patients. All were involved in interpretation of data and statistical analysis. All authors read and approved the final manuscript.
